# Feasibility of ActivABLES to promote home-based exercise and physical activity of community-dwelling stroke survivors with support from caregivers: A mixed methods study

**DOI:** 10.1186/s12913-020-05432-x

**Published:** 2020-06-22

**Authors:** Steinunn A. Olafsdottir, Helga Jonsdottir, Ingibjörg Bjartmarz, Charlotte Magnusson, Héctor Caltenco, Mikko Kytö, Laura Maye, David McGookin, Solveig Asa Arnadottir, Ingibjörg Hjaltadottir, Thora B. Hafsteinsdottir

**Affiliations:** 1 Department of Physical Therapy, Faculty of Medicine, School of Health Sciences, University of Iceland, Stapi við Hringbraut, 102, Reykjavik, Iceland; 2 Faculty of Nursing, School of Health Sciences, University of Iceland, Reykjavik, Iceland; 3 Division of Clinical Services I, Landspitali- The National University Hospital of Iceland, Reykjavik, Iceland; 4 Department of Design Science, Lund University, Lund, Sweden; 5 Department of Computer Science, Aalto University in Helsinki, Espoo, Finland; 6 Department of Computer Science, University of Helsinki, Helsinki, Finland; 7 Nursing Science Department, Julius Center for Health Sciences and Primary Care, University Medical Center Utrecht, Utrecht, The Netherlands

## Abstract

**Background:**

Technical applications can promote home-based exercise and physical activity of community-dwelling stroke survivors. Caregivers are often able and willing to assist with home-based exercise and physical activity but lack the knowledge and resources to do so. ActivABLES was established to promote home-based exercise and physical activity among community-dwelling stroke survivors, with support from their caregivers. The aim of our study is to investigate the feasibility of ActivABLES in terms of acceptability, demand, implementation and practicality.

**Methods:**

A convergent design of mixed methods research in which quantitative results were combined with personal experiences of a four-week use of ActivABLES by community-dwelling stroke survivors with support from their caregivers. Data collection before, during and after the four-week period included the Berg Balance Scale (BBS), Activities-Specific Balance Confidence Scale (ABC), Timed-Up-and-Go (TUG) and Five Times Sit to Stand Test (5xSST) and data from motion detectors. Semi-structured interviews were conducted with stroke survivors and caregivers after the four-week period. Descriptive statistics were used for quantitative data. Qualitative data was analysed with direct content analysis. Themes were identified related to the domains of feasibility: acceptability, demand, implementation and practicality. Data was integrated by examining any (dis)congruence in the quantitative and qualitative findings.

**Results:**

Ten stroke survivors aged 55–79 years participated with their informal caregivers. Functional improvements were shown in BBS (+ 2.5), ABC (+ 0.9), TUG (− 4.2) and 5xSST (− 2.7). More physical activity was detected with motion detectors (stand up/sit down + 2, number of steps + 227, standing + 0.3 h, hours sitting/lying − 0.3 h). The qualitative interviews identified themes for each feasibility domain: (i) acceptability: *appreciation, functional improvements, self-initiated activities* and *expressed potential for future stroke survivors*; (2) demand: *reported use, interest in further use* and *need for follow-up;* (3) implementation: *importance of feedback, variety of exercises* and *progression of exercises* and (4) practicality: *need for support* and *technical problems.* The quantitative and qualitative findings converged well with each other and supported the feasibility of ActivABLES.

**Conclusions:**

ActivABLES is feasible and can be a good asset for stroke survivors with slight or moderate disability to use in their homes. Further studies are needed with larger samples.

## Background

Stroke is one of the main causes of chronic disability in the Western world [1]. Engaging in ongoing exercise and physical activity is important after stroke to maintain and improve physical function [2, 3] and as a method of secondary prevention of stroke [4]. Therefore, exercise and physical activity need to be a lifelong part of the daily life of community-dwelling stroke survivors. Despite this knowledge, community-dwelling stroke survivors are physically inactive [5] and they sit for long periods of time [6]. Lack of motivation and confidence can diminish stroke survivors’ participation in exercise and physical activity after inpatient rehabilitation [7–9], when they need to rely more on themselves and their informal caregivers to continue with exercise and physical activity. At the same time, community-dwelling stroke survivors and their informal caregivers report uncertainty regarding what they can do to maintain and/or improve function at home [10] and might often be in need of practical and emotional support to continue with exercise and physical activity.

In recent years, home-based exercise programmes have been increasingly developed to promote exercise and physical activity among community-dwelling stroke survivors [11–15]. Home-based exercise programmes can result in improved function of stroke survivors, including better balance and more involvement in activities of daily living [16, 17]. Family members and other informal caregivers are able to assist stroke survivors with exercises that are supervised by physical therapists or other members of the rehabilitation team [17–19] and it can be motivating for stroke survivors to do such exercises [20]. Informal caregivers are generally willing to assist with exercise and feel more content if they are able to assist [18, 21]. On the other hand, informal caregivers often lack knowledge and support and they need more education on how they can provide support with exercise and physical activity [22, 23]. Many studies have revealed a need for practical support for stroke survivors and their informal caregivers to help them engage in home-based exercise [10], and recent studies have suggested support could be provided by technical applications [24–27].

Technical applications, such as virtual reality and computer games, can support stroke survivors with home-based exercise [28, 29], encourage them to adhere to the exercises [25, 30–32] and decrease sedentary behaviour [30]. Stroke survivors with mild to moderate residual deficits have been shown to benefit more in terms of functional improvements with use of technical applications than stroke survivors with more severe deficits [31]. Technical applications can offer a variety of repetitive and challenging functional tasks [29, 31] that can encourage plasticity of the brain and enhance motor learning. Stroke survivors are generally willing to use technical applications to assist with home-based exercise [32–34] and many studies have investigated different technical approaches [24, 30, 35, 36]. Virtual reality has been defined as a user-computer interface with real-time simulation and has been shown to increase activity significantly more than conventional therapy [31, 37]. Game-based interventions are thought to be more enjoyable than traditional therapy and have shown to be more effective in improving balance and independence than traditional exercises in stroke survivors [29]. This evidence supports the hypothesis that technical approaches have the potential to be used to promote home-based exercise and physical activity among stroke survivors. Therefore, it is important to continue to develop useable and feasible technical applications for stroke survivors that can be used successfully in their homes.

Based on this background, and as a way to respond to stroke survivors’ and informal caregivers’ needs for home-based exercise and physical activity, an international collaborative project was established to develop ActivABLES. ActivABLES is a modular technological intervention, comprising multiple exchangeable components, to promote home-based exercise and facilitate physical activity engagement of community-dwelling stroke survivors with support from their informal caregivers. The aim of our study is to investigate the feasibility of ActivABLES for community-dwelling stroke survivors and their informal caregivers (hereafter referred to as caregivers) in terms of acceptability, demand, implementation and practicality of the intervention.

## Methods

### Design

**Fig. 1:**
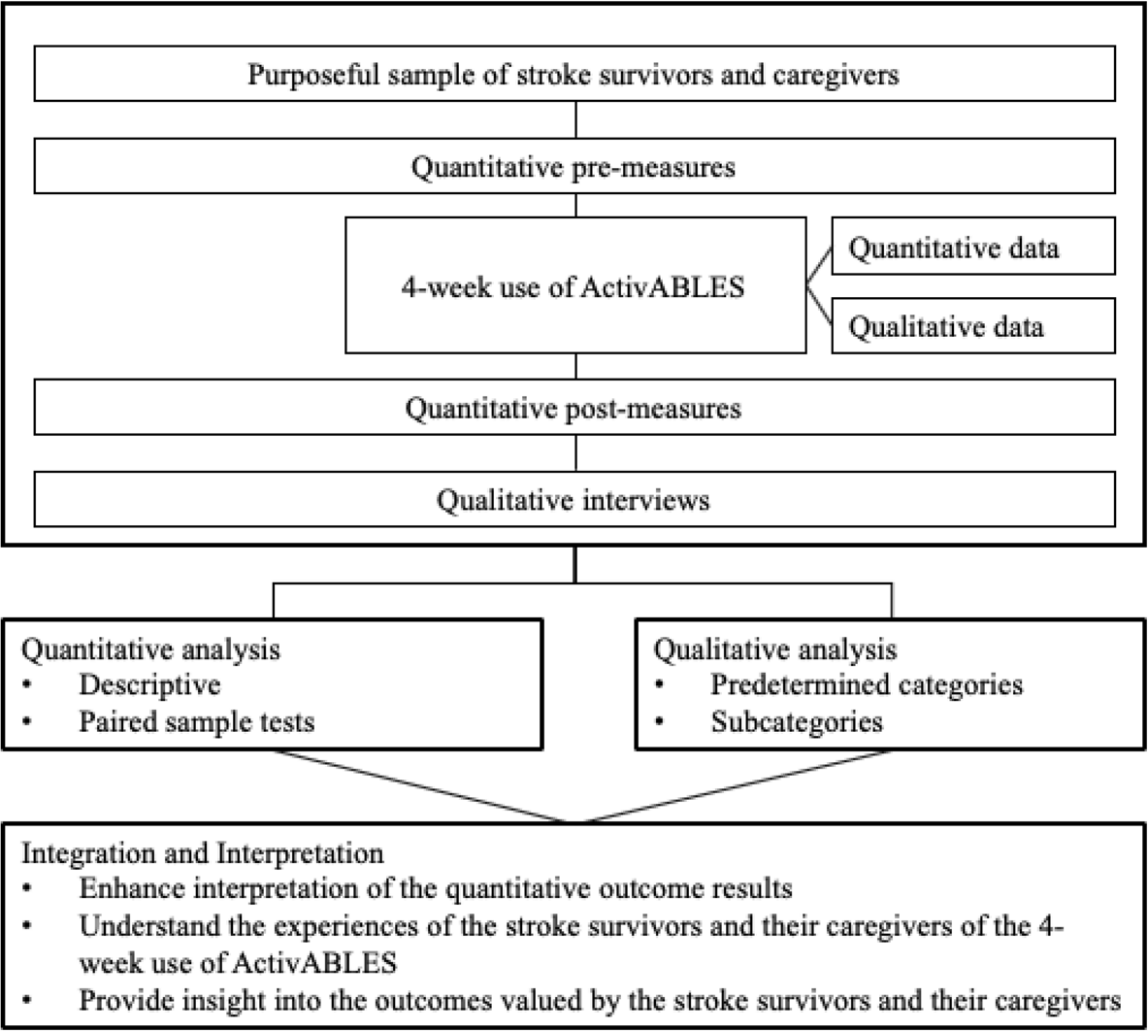
Diagram of the mixed methods study

A feasibility pilot study was conducted using a convergent mixed method design [38], which included concurrent collection of quantitative and qualitative data, as well as independent interpretation of the data and integration to evaluate the feasibility of ActivABLES after a four-week use (Fig. 1). Since ActivABLES includes different tools aiming to improve various outcomes of community-dwelling stroke survivors, the Medical Research Council’s (MRC) framework for development and evaluation of complex health interventions was used to guide the development and testing process [39, 40]. Studying feasibility is an important part of the development and evaluation of complex interventions according to the MRC model [39]. Feasibility was evaluated in terms of acceptability, demand, implementation and practicality, which are four components of the feasibility framework presented by Bowen (2009) [41]. Acceptability assesses how the stroke survivors and their caregivers react to ActivABLES and how suitable, satisfying or attractive they think the tools are. Demand looks at how ActivABLES is used by the stroke survivors and how likely it is they will use the tools in the future. Implementation focuses on the execution, type of resources and factors affecting the implementation of ActivABLES and how the tools can be improved. Practicality assesses how ActivABLES is delivered to stroke survivors and how they manage the use of the tools with regard to resources, assistance/support and circumstances [41].

### Sample and participants

In our study, we used a purposive sampling of community-dwelling stroke survivors and their caregivers. Participants were approached through inpatient rehabilitation clinics and outpatient physical therapy clinics. We included stroke survivors who met the following criteria: older than 18 years of age; at least 4 months since discharge from hospital or inpatient rehabilitation; with slight or moderate impairment defined by a score of 2–3 on the Modified Rankin Scale [42]; with no severe cognitive deficits defined by the Mini Mental State Examination (> 24) [43]; no severe comorbidities or pre-existing conditions affecting function or ability to speak and understand Icelandic. Also included were informal caregivers (hereafter referred to as caregivers), defined as a family member or a close friend in a good relationship with the stroke survivor, older than 18 years of age, and able to communicate and assist the stroke survivor. All the participating stroke survivors and caregivers received verbal and written information about the study, emphasising that participation was voluntary, anonymous and confidential. The participants and the researcher (SAO) signed an informed consent prior to participating in the study.

### ActivABLES

The development of ActivABLES used human-centred design (ISO 9241-210:2010) including elements of participatory design/co-design [44]. Thus, prototype designs were tested iteratively in collaboration with community-dwelling stroke survivors and health professionals during the whole development process. The MRC framework for the development and evaluation of complex interventions further guided the development of the ActivABLES as a healthcare intervention. The development process is described in detail in a separate paper [45]. ActivABLES consists of six tools (Table 1): (1) ActivFOAM with interactive games for balance exercises [46]; (2) Walking STARR, an iPhone application which includes a step counter, activity monitoring and games [47]; (3) ActivBALL to exercise motor control of the wrist and shoulder and the grip strength; (4) ActivSTICKS to exercise motor control of the shoulder and upper body [48]; (5) ActivLAMP which gives feedback on daily progress in one exercise, and (6) ActivTREE which gives feedback on daily progress in up to three exercises. All the ActivABLES tools give an instant feedback in the form of sound and/or light which is intended to strengthen the motivation to exercise.

**Table 1:** Prototypes of ActivABLES tested in the feasibility study

	Description	Purpose
**ActivFOAM**	A foam balance mat with pressure sensors that gives individually tailored visual and audio feedback on weight shifting and center of mass while standing. The mat is connected to a tablet which is positioned in front of the user. Three games and different forms of audio feedback can be selected from the tablet.	To exercise balance and weight-bearing in a standing position.
**Walking STARR**	An application for iPhone which records steps and walking time. The idea is to simulate taking the dog for a walk. Games include having to stop to let the dog pee and eat. Finishing games allows the user to collect stars.	To motivate and provide feedback on progress of walking.
**ActivBALL**	A soft ball to exercise motor control of the arm and develop grip strength. The ball is connected to a tablet which is positioned in front of the user and which can be pre-programmed for individually tailored sets of exercises. The range of motion and pressure detected while squeezing can be adjusted for each user. The tablet gives feedback by counting the repetitions. The ball can be used to exercise: 1) forearm pronation/supination, 2) dorsiflexion and palmar flexion of the wrist, 3) external/internal rotation of the shoulder, 4) flexion and extension of the fingers while squeezing.	To exercise the motor control of the hand and forearm
**ActivSTICKS**	Two sticks linked together forming an angle from 0° to 180°. The sticks are connected to a tablet which is positioned in front of the user and which can be pre-programmed for individually tailored sets of exercises. The range of motion detected, and resistance can be adjusted for each user. The tablet gives feedback by counting the repetitions. The sticks can be used to exercise: 1) abduction and adduction of the shoulder, 2) flexion of the shoulder, 3) elbow flexion and extension, along with coordination of the left and right arms while doing “scissors”, 4) rotation of the upper body.	To exercise the motor control of the shoulder and upper body.
**ActivLAMP**	A lamp that that gradually brightens in connection with exercises or physical activities. The lamp is connected to a tablet and can be connected to any of the above exercise tools.	To motivate and provide feedback on progress of exercises or walking.
**ActivTREE**	A tree that has three branches that gradually brighten in connection with exercises and physical activities. The tree is connected to a tablet and can be connected to any of the above exercise tools. Each branch represents a different tool and they all share the same trunk.	To motivate and provide feedback on progress of exercises and walking.

### Data

Quantitative and qualitative data were collected prior to, during and after the four-week use of ActivABLES (Fig. 1). Data from functional measures, questionnaire, motion detectors, digital servers, adherence diaries and semi-structured interviews were used to evaluate the feasibility of ActivABLES.

The mobility and functional progress of the stroke survivors were evaluated before and after the four-week use of ActivABLES using the following measures:

- Static and dynamic balance was measured with the *Berg Balance Scale* (BBS) [49] which consists of 14 static and dynamic activities of varying difficulty. Each item gives a score of 0–4 and the maximum score is 56 which indicates good functional balance. The psychometric properties of the BBS for stroke survivors show good and excellent results [50–53].
- Balance self-efficacy when performing activities was measured with the *Activities-Specific Balance Confidence Scale* (ABC) [54]. ABC is a 16-item self-report measure in which participants rate their balance confidence for performing activities on a scale of 0–100%. The psychometric properties of ABC for stroke survivors show good and excellent results [55, 56].
- General mobility was measured with the *Timed-Up-and-Go* (TUG) [57]. In TUG, the participant stands up from a chair, walks a distance of three meters, turns around, walks back to the chair and sits down. The time required to perform the TUG is recorded using a stopwatch. The psychometric properties of the TUG for stroke survivors show good and excellent results [58, 59].
- Functional lower limb muscle strength was measured with the *Five Times Sit to Stand Test* (5xSST) [60], which measures the time required to perform the 5xSST, using a stopwatch. The psychometric properties of the 5xSST for stroke survivors show good results [61].
- Arm and hand function were measured with the *Box and Block Test* (BBT) [62]. In the BBT, the participant moves as many cubes between boxes as possible in 1 min. The psychometric properties of the BBT for stroke survivors with arm paresis show good and excellent results [63, 64].

Motivation to exercise was measured with the *Behaviour Regulation Exercise Questionnaire 2* (BREQ-2) [65]. The BREQ-2 is a 19-item questionnaire, where each question is answered on a five-point Likert scale ranging from 0 (not true for me) to 4 (very true for me). BREQ-2 was developed to assess exercise behaviour based on the self-determined theory (SDT), which is a popular framework to assess motivation in exercise psychology [66]. In the SDT various forms of motivation represent different ways in which behaviour can be regulated, ranging from completely non-self-determined to completely self-determined regulation. The BREQ-2 has five subscales: (i) amotivation (lack of any intention to engage in exercise), (ii) external regulation (engaging in exercise only to satisfy external pressures or to get externally imposed rewards), (iii) introjected regulation (self-imposed pressures to avoid guilt or maintain self-esteem), (iv) identified regulation (accepting exercise as an important factor to achieve personally valued outcomes) and (v) intrinsic regulation (taking part in exercise for the enjoyment and satisfaction of it) [66]. In line with SDT, amotivation and external and introjected regulation address non-self-determination with scoring of 0–44, while identified and intrinsic regulation address self-determination with scoring of 0–32 [67]. Lower scoring of non-self-determination and higher scoring of self-determination is positively linked with adaptive health behaviour [68] indicating that people are more aware of the outcomes of exercise and feel more committed to it [69]. The psychometric properties of the BREQ-2 have been investigated in a sample of healthy people indicating good construct validity [66, 70, 71] and have been used in different patient groups [67, 72, 73].

The actual use of ActivABLES was evaluated by connecting all the ActivABLES tools to a server which collected digital data on the frequency and length of use of ActivABLES.

Data on sedentary, upright and ambulatory activities were collected with *ActivPAL motion detectors* (PAL Technologies Ltd., Glasgow, UK). The stroke survivors wore the motion detectors around their non-affected thigh for 7 days (24 h) at three different time points; a week prior to the start of the four-week period of ActivABLES, midway through the study and a week after the four-week period. The data generated represents a 24-h summary of time spent in sitting/lying and standing positions and taking steps, number of transitions from sitting to standing and number of steps taken. Motion detectors have been used in many studies to explore physical activity [74, 75] in stroke survivors [76].

The caregivers were asked to filled in the adherence diaries during the four-week use of ActivABLES, which provided both quantitative and qualitative data. The adherence diaries had a format for each of the ActivABLES tool including questions on the frequency and length of use (in minutes), which exercises were done with each tool, the execution of the exercises and the need for support and motivation. A Borg scale [77] was used to assess perceived exertion, which evaluated the intensity of the exercises (0 indicated no exertion and 10 indicated much strain) and experienced execution, which evaluated how they managed using the tools (0 indicated “impossible to use” and 10 indicated “very useable”). In addition, there was an empty place in the diaries where the caregivers were asked to write down their thoughts and comments on their experience of the exercises and the feasibility of using ActivABLES.

Qualitative data was collected with semi-structured interviews [78] which were conducted separately with each stroke survivor and their caregivers after the four-week use of ActivABLES, to gain deeper understanding of how they experienced the feasibility of ActivABLES. The interview guides included questions which focused on the feasibility of ActivABLES in terms of acceptability, demand, implementation and practicality (Table 2).

**Table 2:** Interview guides for stroke survivors and informal caregivers

Stroke survivors	Informal caregivers
1. Why did you decide to participate in this research?	1. How have the exercise been going over the last 4 weeks? (Ask about all the tools)
2. Did you exercise at home before this research? Why / Why not?	2. Has the stroke survivor been following the exercise protocol through the whole period of 4 weeks? Do you feel his/her motivation has changed over the time? How?
3. What is your overall experience of doing the exercise over last 4 weeks?	3. Did you need to encourage the stroke survivors to exercise using the tools? Over the whole period?
4. Have you been able to follow the exercise program over the period? Did your motivation change over time?	4. Did you need to assist the stroke survivor with the exercises or using the tools? If yes, how? Please describe further?
5. Did you feel the tools encouraged you to continue?	5. Were there exercises/tools that the stroke survivor did liked more or less than others? What was it about the exercises/tools that the stroke survivor liked or disliked?
6. What exercise/tool did you like the most / the least? How/why? Please describe further	6. Were there exercises/tools that the stroke survivor felt were more challenging / less challenging? If yes, please describe further?
7. What exercise/tool did you feel was most challenging / least challenging? How/why? Please describe further	7. Do you think the general physical activity of the stroke survivor has changed over the last 4 weeks? Has he/she been doing something on a daily basis that he/she had not been doing recently? Please describe further.
8. Do you think your general physical activity has changed over last 4 weeks? Have you been doing something more/less on a daily basis than before?	8. Do you think these tools can be useful for the stroke survivor permanently? Why? / Why not?
9. Do you feel like you could continue to use these tools for an unlimited time? Why? / Why not?	9. What is your overall experience of using the tools? – Is there something that needs to be changed?
10. Do you think these tools could be useful in doing exercises at home – to maintain / improve your health? Why? / Why not?	
11. What is your overall experience of using the tools? – Is there something that needs to be changed? – How/why?	

### Procedure

**Fig. 2:**
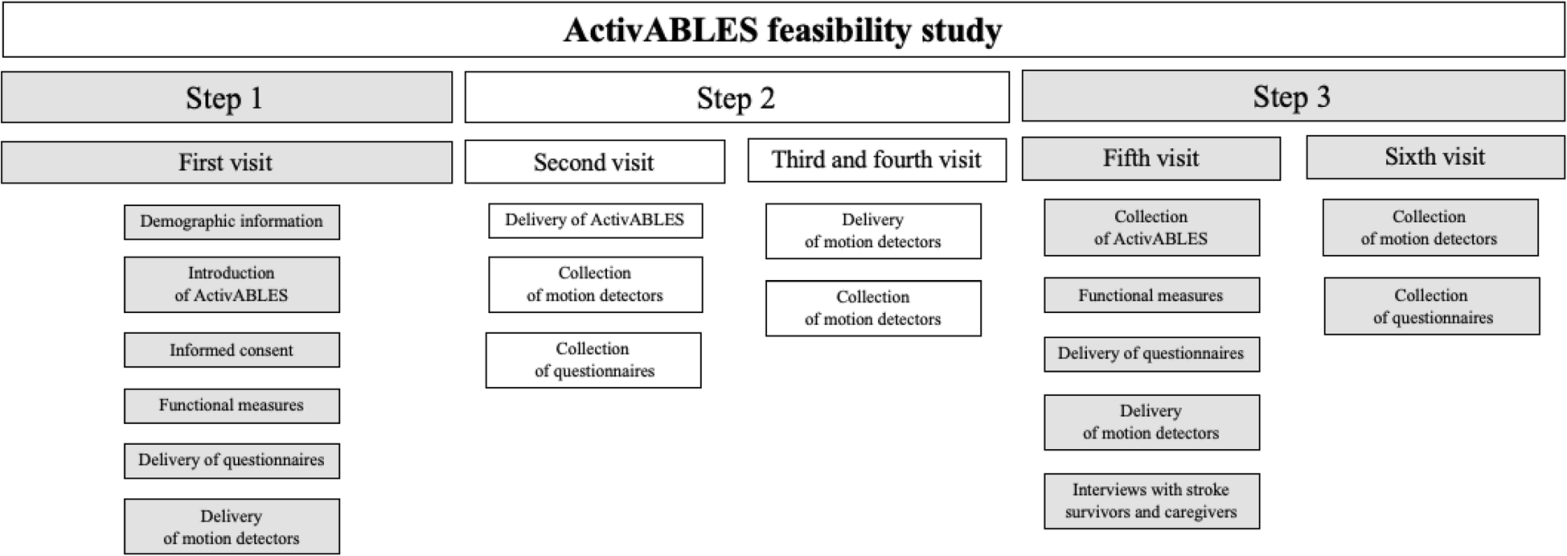
Procedure of data collection

Data was collected over a six-week period, which included a four-week use of ActivABLES (Fig. 2). Two researchers, a physical therapist (PT) (SAO) and a registered nurse (RN) (IB) collected the data in three steps:

#### Step 1. Pre-test measures

A week prior to the four-week use of ActivABLES (week 1), the two researchers (SAO and IB) visited the stroke survivor and his/her caregiver. Detailed information about the study was given and ActivABLES was introduced to the participants. Demographic and clinical data were collected, including information on time since stroke and the side of hemiparesis. The stroke survivors were also asked about indoor use of walking devices and all participants were asked if they used tablets/computers on a daily basis. Baseline functional measures were carried out, and two self-report questionnaires (ABC and BREQ-2) were left with each stroke survivor to answer with assistance from caregiver if needed. A motion detector was attached to the stroke survivor’s affected leg to wear for 7 days.

#### Step 2. ActivABLES

Together with the stroke survivors and their caregivers, the PT selected relevant ActivABLES tools to be used, based on the pre-tests and the needs of each stroke survivor. Each participant was assigned at least one exercise tool (maximum of three) and one feedback tool (maximum of two). Both researchers, along with technicians, visited each stroke survivor at his/her home with selected ActivABLES tools and delivered an interactive training session on their use along with written guidance on how to use them. The stroke survivors were asked to use the selected ActivABLES tools at least five times per week for 4 weeks. The recommended daily use was determined on an individual basis in agreement with each stroke survivor, ranging from 10 to 30 min. The stroke survivors were encouraged to gradually increase the duration of use, with the aim of exercising for 30 min per day. Caregivers were involved and gave practical and social support which could include assistance with using the tools, thereby ensuring safety and providing encouragement during the four-week period. Researchers provided adherence diaries and gave information to the caregivers on how to fill them in. The participants were encouraged to contact the researchers via phone or email, if problems occurred. One of the researchers visited the participants after approximately 10 days of using ActivABLES (third visit) and attached a motion detector to the stroke survivor to wear for the next 7 days. This was picked up a week later (fourth visit).

#### Step 3. Post-test measures

After the four-week use of ActivABLES, the researchers visited each stroke survivor (fifth visit) and repeated the functional measures and conducted semi-structured interviews with the stroke survivors and their caregivers about their experiences of using ActivABLES. The PT carried out the functional measures while the RN documented the results of the measures to avoid performance bias. The PT interviewed the stroke survivors while the RN interviewed the caregivers. The interviews were recorded and transcribed verbatim.

### Data analysis

Quantitative data, including the demographic data, functional measures, digital data and data from the adherence diaries, were recorded in Excel and transferred into *jamovi software*, version 0.9 (Retrieved from https://www.jamovi.org, 2018). Descriptive statistics were used to analyse quantitative data, including medians and interquartile range for continuous data. Imputation was used to approach missing data in BREQ-2, using predictive mean matching [79]. The data was imputed with the statistical package mice in *R, statistical software* (The R Foundation for Statistical Computing, Vienna, Austria). Qualitative data were analysed using direct content analysis [80] and themes identified from the data based on the four domains of feasibility: acceptability, demand, implementation and practicality as suggested by Bowen et al. (2009) [41]. One researcher (SAO) identified themes according to the domains and discussed these with co-authors until agreement on the content was reached. Quotes related to the identified themes were translated from Icelandic to English. Quantitative and qualitative data were then integrated by looking for common concepts across the data, comparing the data and examining any (dis)congruence in the findings.

## Results

### Participants

A total of 20 individuals took part in the study, including 10 stroke survivors and 10 caregivers. The stroke survivors were five women and five men, with the median age of 72 years (range 55–79 years), and the time since stroke ranging from 5 months to 30 years. Six stroke survivors had left hemiparesis and four had right hemiparesis. Four stroke survivors used assistive walking devices. Eight stroke survivors went to physical therapy every week. Ten caregivers were included, seven women and three men, who were all family members, with the median age of 69 years (range 28–80 years). Five caregivers were retired, four were employed and one was unemployed due to disability. Six stroke survivors and seven caregivers used a personal tablet/computer on a regular basis (Table 3).

**Table 3:** Characteristics of all participants

Stroke survivors					Informal caregivers		
age	time since stroke	side of hemiparesis	walking device inside	tablet/computer use on daily basis	age	occupation	tablet/computer use on daily basis
63	23 years	left	no	yes	68	working part-time	yes
55	9 months	right	no	no	28	unemployed	yes
71	15 months	left	yes, a cane	yes	72	retired	no
79	5 months	right	no	yes	79	retired	no
66	26 months	right	no	yes	66	working part-time	yes
74	19 months	left	yes, a cane	no	70	retired	yes
67	8 months	left	no	yes	58	working full-time	yes
73	30 years	left	yes, a crutch	yes	51	working full-time	yes
78	4 years and 3 months	left	yes, a crutch	no	79	retired	yes
72	14 months	right	no	no	80	retired	yes

All the stroke survivors were given the ActivFOAM for balance exercises to use for the 4 weeks, two received the ActivSTICKS for exercising the upper arms, and two were given the ActivBALL for exercising the arm and hand. Four stroke survivors received the walking application to record their step counts while walking, six were given the ActivLAMP and five received the ActivTREE for visual feedback.

### Quantitative findings

All the stroke survivors took part in the functional pre-tests but only nine took part in the post- tests since one stroke survivor was hospitalised for some days during the four-week period (Table 4.). Seven stroke survivors, who took part in both pre and post measures of function, improved in two or more measures. The median of the functional measures showed improvements in all tests. The results of BBS changed from 43.5 to 46.0 and scoring of the ABC-Scale improved from 55.5 to 56.4. The participants needed 4.2 s less to finish TUG and were 2.7 s faster to finish 5xSST. The data from the motion detectors showed more physical activity during and after the intervention, with a higher median in the number of standing up/sitting down, and steps and hours standing, and fewer hours spent sitting/lying. The results from the BREQ-2 for motivation to exercise showed higher self-determined motivation than non-self-determined motivation to exercise in both pre and post measures, indicating that the stroke survivors valued the benefits of exercise.

**Table 4:** Quantitative measures

	pre-test^a^		halftime of the intervention^a^		post-test^a^		change in score^b^
Berg Balance Scale (0–56)	43.5	(39–47.3)			46.0	(43.0–48.0)	↑ 2.5
ABC-Scale (%)	55.5	(39.1–58.8)			56.4	(46.0–67.2)	↑ 0.9
Timed-Up-and-Go (sec)	20.1	(17.6–21.3)			15.9	(12.5–19.2)	↑ 4.2
Five Times Sit to Stand (sec)	20.9	(17.4–27.0)			18.2	(16.7–20.3)	↑ 2.7
Box and Block Test (no blocks)	33	(31–35)			33	(32–34)	0
Data from motion detectors							
standing up/sitting down (times/day)	47	(32–50)	48	(46–50)	49	(42–56)	↑ 2
number of steps (per day)	1836	(1706–2636)	2469	(1707–3036)	2063	(1724–2998)	↑ 227
standing (hours/day)	2.3	(1.7–3.2)	2.6	(2.0–3.1)	2.6	(1.8–3.1)	↑ 0.3
sitting/lying (hours/day)	21.3	(20.4–22.4)	21.4	(20.8–22)	21.0	(20.6–22.3)	↑ -0.3
Behaviour Regulation Exercise Questionnaire							
non-self-determined motivation (0–44)	9	(8.3–12.8)			8.5	(8.0–9.75)	↑ 0.5
self-determined motivation (0–32)	28	(24.3–29.5)			26	(25.3–26)	↓2.0

^a^median (1st and 3rd quartile)

^b^ the arrows indicate if the change is positive (↑) or negative (↓)

According to the digital servers, seven stroke survivors used ActivABLES for the recommended 5 days a week for the 4 weeks with the median use of 23 days, (range 5–27 days). Four of the adherence diaries were thoroughly filled in, whereas six diaries gave reports for only a limited number of days*.* The data from the four diaries on the number of days that the tools were used, correlated with the data on the number of days reported on the digital servers. However, more use was reported on the number of minutes spent exercising in the adherence diaries when compared to data on the digital servers. The average daily use per participant reported in the diaries was in the range of 14–48 min, whereas on the servers the average daily use range was nine to 28 min.

The stroke survivors and/or their caregivers called the researcher 19 times in total, to ask for advice and/or report technical difficulties during the four-week period. On nine occasions restarting the tablet was enough to resolve the issue and twice the caregivers were able to take care of some minor configurations. On seven occasions, additional support was needed and two phone calls were to report accidents with one of the tools and a tablet which fell on the floor and broke.

### Qualitative findings

**Fig. 3:**
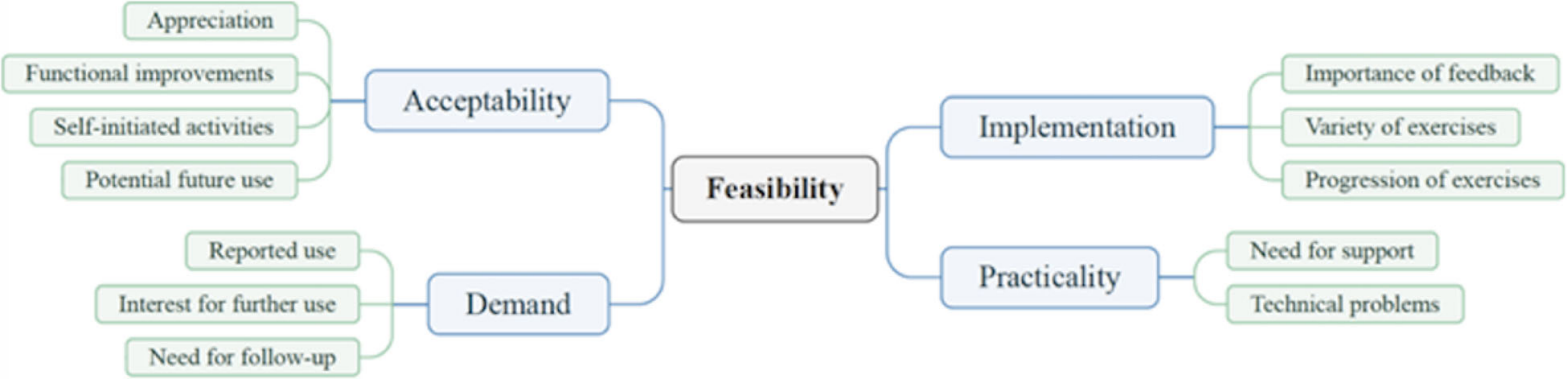
Themes identified in line with feasibility domains

The feasibility of ActivABLES was described by the participants in terms of four feasibility domains: acceptability, demand, implementation and practicality. Twelve themes emerged from these domains which further explicate the domains and quotes illustrate the themes within each domain (Fig. 3).

#### Acceptability

Four themes were identified that illustrate the acceptability of ActivABLES: (1) appreciation, (2) functional improvements, (3) self-initiated activities and (4) potential use for future stroke survivors (Fig. 3).

##### Appreciation

Both stroke survivors and caregivers expressed appreciation for being offered an opportunity to take part in the development of ActivABLES to promote home-based exercise and physical activities.

*But I think this is really great, I would have liked to get this much sooner...(Stroke survivor)*

*I think this is an excellent initiative and I just hope that they will be able to refine this and put it into use. (Caregiver)*

Functional improvements: Most stroke survivors and their caregivers described improvements in functioning. Few did not notice improvements including the man who was hospitalised and could not use ActivABLES as recommended, as well as those who had stroke long time ago.

*I think my walking is better, at least they [in physical therapy] say that my gait has improved. (Stroke survivor)*

*I don’t know if it is because of this but she is doing better when she is walking, she does not use the walker inside anymore, She is walking around so her mobility is getting better. (Caregiver)*

*Self-initiated activities* were identified when participants described activities they had not recently taken part in. They stroke survivors described increased motivation to engage in self-initiated activities such as walking indoors without using a cane/crutch and washing the floor. They were also more aware of using the affected arm. In addition, the caregivers also described noticing self-initiated activities by the stroke survivors.

*Yes, for example [the exercises] motivated me to try to take a shorter stroll without a crutch, and to take the posh-stick as I call it, (my cane) to practice with it. It's obviously at the top of the wish list to walk with a cane. (Stroke survivor)*

*He gets things himself a lot more, he is not calling for me all the time, he just stands up and goes to the freezer and gets things. In this respect I feel there is a big difference, he is helping himself a lot more. (Caregiver)*

##### Potential use for future stroke survivors

All participants described ActivABLES to have potential use for future stroke survivors.

*This can do so much more than I expected when I first saw it last year or the year before. I think this ActivABLES has a potential, especially for younger people or people right after the stroke. It is just so important to move around as much as you can with help from professionals. (Stroke survivor)*

*Absolutely, especially the balance exercises for people who haven’t too much paresis or are not so insecure when walking. The ball could also be useful for all stroke survivors, even though you are in wheelchair. (Caregiver)*

#### Demand

Three themes that illustrated demand were identified: (5) *reported use,* (6) *interest in further use* and (7) *need for follow-up* (Fig. 3).

Most stroke survivors and their caregivers described having tried to use ActivABLES at least five times a week over the four-week period, but some said they quit few days earlier due to technical issues, mostly connection issues between the tablet and the tools.

*I have been using the tools as conscientiously as I can every single day but maybe for a shorter time than I would have liked some days. (Stroke survivor)*

*I think he has been doing very well with this, he has dedicated himself to using it and he is interested in it. (Caregiver)*

##### Interest in further use

The stroke survivors said they would be interested in further use of ActivABLES themselves. The caregivers also thought “their” stroke survivor would be interested in further use.

*It would be good to get a plan of exercises to follow; something like this, for the balance. (Stroke survivor)*

*Yes, these movements he is doing, he likes it and it seems to be doing him good. (Caregiver)*

##### The need for follow up

The stroke survivors and caregivers emphasised the need for follow-up services and said that ActivABLES might have potential as a part of this kind of service if supervised by a rehabilitation professional.

*I think that current follow-up services for people like me who have had a stroke are not good enough. - This is kind of a follow-up [like ActivABLES] is lacking. (Stroke survivor)*

*What she needs is more physical activity; like if somebody would come and take her out for a walk. (Caregiver)*

#### Implementation

Three themes that illustrated implementation were identified from the interviews: (8) the *importance of feedback,* (9) *variety of exercises* and (10) *progression of exercises* (Fig. 3).

##### Importance of feedback

Both stroke survivors and caregivers said that the feedback was very important while doing the exercises. The stroke survivors also mentioned how many points they had scored and their enthusiasm for competing for more points. Having a target to compete for was also described by the caregivers. The visual feedback from ActivTREE and ActivLAMP were helpful in this regard.

*I once made it up to 100. [Yes, good for you, well done]. Yes, usually I went up to 20 or 30, then I was finished. But I was so upbeat that day that I went up to 100, I thought it was great. (Stroke survivor)*

*I believe the feedback is good . . . You know, being able to fill the tree completely. I realise it is so motivating to be able to do that, and to keep going and do a little more, or even better. - He was happy when the right branch of the tree became fully lit. Yes, it made him happy. (Caregiver)*

##### Variety of exercises

The stroke survivors thought the exercises lacked variety and would have liked to have more diversity in the types of them. Their caregivers agreed with these sentiments.

*The ActivFOAM could have a more diverse list of games, such as Escape. It is sometimes fun, it was particularly fun at the beginning but then it became, like, just boring*. *(Stroke survivor)*

*The interest decreased a little, yesterday or the day before yesterday, she talked about it, saying that the variety, it was missing a bit. (Caregiver)*

##### Progression of exercises

Some stroke survivors said they had progressed with the exercises and that they made attempts to make them more challenging. In the adherence diaries, the stroke survivors rated the exercises as more difficult at the beginning (5–8 out of 10). During the final days of the study they had become less difficult (1–5 out of 10).

*They [the games] should not be too hard, but something that everybody can do as an exercise. And then you could make them more difficult for progression. (Stroke survivor)*

#### Practicality

Two themes that illustrated practicality were identified: (11) *need for support* and (12) *technical problems* (Fig. 3).

##### Need for support

Only few caregivers described occasional encouragement or reminders for doing the exercises. One caregiver said she physically had to assist her husband with the exercises. Others described assisting such as with turning on ActivABLES and/or charging the tablet. The stroke survivors described they were almost independent as regards using ActivABLES and doing the exercise.

*I think it might have happened about three times, like “well now, let’s hurry up with this” [caregiver said]. Yes, three times or something, but no more. Otherwise she was always just, she called me when she was done to tell me that she was so happy, you see. (Caregiver)*

##### Technical problems

There were technical problems in relation to the use of ActivABLES. All participants had a problem at some point in time during the four-week period. Sometimes it was enough to restart the tablet. Those who were familiar with using tablets or computers were aware of that and had already tried that before contacting the researchers. On one occasion, the ActivBALL became dysfunctional after falling accidentally on the floor and it was not possible to fix or replace it. Some participants had a tablet that had the same input for charging and for connecting the ActivFOAM and on one occasion, the tablet fell on the floor and broke while a stroke survivor was plugging in the cables. This happened during the last week of the four-week period. The tablet was still useable, but the crack irritated the user and did affect use of the tablet. Some stroke survivors reported frustration when dealing with the technical issues.

*It has challenged my patience, a bit (silence). [Interviewer: Was it mainly due to technical problems or?] Well, just yes, because the devise apparently didn't work completely, despite ones wishes. (Stroke survivor)*

*There were some technical difficulties and then, just her physical fatigue, physical and mental fatigue caused annoyance and a lack of willingness to do anything (silence) -the exercises. (Caregiver)*

#### Integration of quantitative and qualitative findings

The integration of the quantitative and qualitative data is summarised in Table 5. There was congruence in all components of the feasibility framework both between the quantitative and qualitative data and generally also between the stroke survivors and the caregivers. Both accepted the usability of ActivABLES. The stroke survivors also improved in function and physical activity after the four-week use of ActivABLES. The stroke survivors had tried to use ActivABLES for at least 5 days per week. Participants — stroke survivors and caregivers — were in agreement on the potential of ActivABLES for further use in the homes.

**Table 5:** Integration of quantitative and qualitative findings

Feasibility domains	Quantitative results			Qualitative themes	Integration
**Acceptability**	Measure	Change in median from pre to post		Functional improvements	
	BBS (score)	43.5–46	↑ 2.5	Stroke survivors reported improvements in function. Caregivers reported improvements in function of their stroke survivors.	The quantitative functional measures confirm the experience of the participants of improved function.
	ABC (score)	55.5–56.4	↑ 0.9		
	TUG (sec)	20.1–15.9	↑ 4.2		
	5xSST (sec)	20.9–18.2	↑ 2.7		
	BBT (score)	33–33	0		
	Motion detectors:			Self-initiated activities	
	Standing up /sitting down	47–49	↑ 2	Stroke survivors described increased motivation to engage in self-initiated activities.	The quantitative data from the motion detectors suggest that the stroke survivors were more mobile which might indicate they engaged in more activities.
	Number of steps	1836–2063	↑ 227		
	Standing (hours/day)	2.3–2.6	↑ 0.3		
	Sitting/lying (hours/day)	21.3–21.0	↑ 0.3		
**Demand**				Reported use	
	Use according to digital servers			Stroke survivors and their caregivers reported use of ActivABLES at least five times a week.	The quantitative data from servers and diaries were congruent with each other while reported use in the interview tended to be more than from the servers and diaries.
	• Seven stroke survivors used ActivABLES for the recommended five days a week.				
	Use according to adherence diaries				
	• Median use 23 days.				
	Measure	Change in mean from pre to post		Interest in further use	
	BREQ-2: Self-determined motivation	28–26	↓ 2.0	Stroke survivors reported interest in further use. Caregivers thought their stroke survivor would be interested in further use.	The quantitative results from BREQ-2 does only partially support the qualitative results on interest in further use.
	Non-self-determ motivation	9–8.5	↑ 0.5		
**Implementation**				Progression of exercises	
	In the adherence diaries, the stroke survivors rated the exercises as more difficult in the beginning (5–8) and less difficult (1–5) during the last days of use.			Stroke survivors reported they had progressed with the exercises, making them more challenging	In the diaries, the stroke survivors report the exercises as being less difficult, which is convergent with what they reported in the interviews. Progression should lead to at least the same level of difficulty.

Although technical problems were frequent when using ActivABLES, the stoke survivors only needed minimal assistance from the caregivers.

## Discussion

The feasibility of ActivABLES was evaluated in a mixed methods study in terms of acceptability, demand, implementation and practicality among 10 stroke survivors. Quantitative and qualitative results were integrated after data analysis to gain a thorough understanding of the feasibility of ActivABLES [38]. The main findings show that both stroke survivors and caregivers found ActivABLES to be feasible for community-dwelling stroke survivors with slight or moderate impairments to use for exercise and physical activity with support from their caregivers. These results encourage the researchers to proceed with further development of the prototypes.

### Acceptability

In our study, the stroke survivors and their caregivers did appreciate the idea of ActivABLES and believed that ActivABLES could be useful and beneficial for themselves as well as for future stroke survivors. Most of the stroke survivors showed improvements in functioning although they only used ActivABLES for the limited time of 4 weeks. Still, it is important to note that most of the stroke survivors had physical therapy once or twice a week during the 4 weeks they were using ActivABLES. Therefore, we do not know how much value ActivABLES had as regards their functional progress.

The outcomes used in our study were chosen to reflect physical function trained while using ActivABLES. Those who did worse on these outcomes at baseline improved more than those who had higher score. Training effects of exercise can appear as soon as after 1 week, especially if the person is inactive, but the effects are considerably greater with regular exercise for several months [81]. Although most of the cortical reorganisation in the brain takes place in the first 6 months after a stroke [82], there is a growing evidence on stroke survivors improving their function in the chronic phase of stroke, well beyond the first 6 months [83]. All but one stroke survivor in our study were in the chronic phase of stroke (> 6 months since stroke), and this person was actually the only stroke survivor who did not show improvements in any of the functional measures. The two stroke survivors that had their strokes more than 20 years ago were, however, less interested in using ActivABLES but both indicated they would have liked to have had something like this in the earlier phase of stroke. Still, both of them did improve their function in three of the functional measures.

Increased duration of exercise can improve function in stroke survivors [84, 85] and therefore it is important to motivate stroke survivors to engage in exercise. Most of the stroke survivors in our study met a PT once or twice a week (individual or group session) and most of them remained inactive between the physical therapy sessions. The stroke survivors in our study seemed to be very inactive when compared with community-dwelling stroke survivors in international studies [5, 86] and are far from meeting the guidelines for physical activity [81]. According to a review conducted in 2017 [86], the average number of daily steps among community-dwelling stroke survivors in the chronic phase was 4078, whereas the range of daily steps taken by the stroke survivors in our study was 1706 steps prior to the intervention to 3036 during the intervention. The average time spent walking daily was 30 min in our study but 88 min in the review [86]. In another review, the average number of daily steps of stroke survivors ranged between 1389 and 7379, and hours standing, or walking ranged from 2.7 to 4.5 h per day [5]. In our study, the average daily standing and walking hours were 2.3. Some of these differences could possibly be explained by the fact that different motion detectors were used in the studies [86]. Still, there are indications that the stroke survivors did increase their physical activity after the four-week use of ActivABLES.

### Demand

Data on reported use were obtained from the interviews, the digital servers and the adherence diaries, all showing that most of the stroke survivors followed the instructions about the daily use of ActivABLES and used it at least 5 days a week. These results of compliance compare well with the findings of other studies investigating the use of technical applications for home-based exercise [30, 87, 88]. Reported use in our study was different, where the digital servers showed much less use in minutes than reported in the interviews and in the adherence diaries. It is well known in research that people tend to overestimate their physical activity [89]. Still, we believe the reports of use as described in the qualitative findings of the interviews and diaries are reliable because they generally agree with the days and the minutes reported in the diaries. Stroke survivors need to stay physically active to maintain their function, but research has shown they are physically inactive and sit for a prolonged time [5, 86, 90]. According to guidelines for prescribing physical activity to stroke survivors, they should exercise their balance and do some strength and functional exercise one to three times per week and walk or do some aerobic activities for 10–60 min two to five times per week throughout life [81]. The results of BREQ-2 showed that the stroke survivors scored high in self-determination at the beginning. ActivABLES did not change the motivation to exercise which was measured with BREQ-2, although the data demonstrate a tendency in a positive direction towards increased self-determination. A systematic review revealed that different methods and lengths of time were needed to change motivation, depending on how motivated individuals were at each time [91]. There was much missing data in the answers to BREQ-2, which may have affected the outcomes. A larger sample is needed to explain whether ActivABLES can increase motivation for exercise and physical activity.

### Implementation

The spontaneous feedback from ActivABLES is thought to be important, both the direct feedback on the tablet for each exercise as well as the feedback for the whole day given by ActivLAMP and ActivTREE. These results are in line with other studies, showing the importance of feedback in terms of personalised goals and activities [25, 92]. At the same time, the stroke survivors found it important to have more variety in game-based exercises to make them both challenging and engaging to them [34, 93]. The results of our study are partly in line with the findings of a meta-analysis from 2018 [35], where interactive games were shown to be effective in improving functional balance of stroke survivors, measured with BBS, but not effective in improving mobility, measured with TUG, like in our study. Enjoyment of exercise motivates stroke survivors to adhere to exercise and physical activity [88] and more variety is likely to increase enjoyment. One stroke survivor in our study was quite active already, aside from ActivABLES use, and followed his activity using an Apple Watch and he did not find a use for the collective feedback given by the ActivLAMP.

### Practicality

All participants agreed there was not much need for support or assistance and stroke survivors were generally self-sufficient with the exercise. The caregivers were willing and able to help and were glad to have a resource to use in their homes to increase the physical activities of their loved one. ActivABLES was easy to handle for the stroke survivors with slight or moderated impairments and they generally did not need assistance, except at the very beginning. As can be expected, some participants experienced technical problems which caused some frustrations. This, however, is always an issue when developing technological prototypes due to their more experimental nature, but it emphasises the importance of having tools that are easy to use and are uncomplicated. This might explain why some stroke survivors did not show full compliance with the recommended use of the ActivABLES tools.

#### Limitations and strengths

Among the limitations of the study are the small sample size and a the lack of a control group which limits the generalisation of the study results. In addition, the participants reported different technical problems when using ActivABLES, which is inherent in a study like this. The tools were technological prototypes, and thus somewhat fragile and vulnerable to minor tumbling. Only four adherence diaries were filled in properly, indicating that more emphasis and/or support from the researchers might have been needed on the importance of documenting the use of the tools properly. The participants may also have become tired of keeping the diaries resulting in less thorough reporting. Moreover, there were missing data in the motion detectors and the self-report questionnaires. Lastly, the researchers who conducted the interviews with participants were known to the participants and may have elicited answers that were desirable rather than an accurate reflection of the actual experience. However, to minimize the risk of bias the researchers emphasised the need for negative as well as positive feedback on using ActivABLES.

Our study had various strengths that need to be emphasised. With an innovative technical intervention like ActivABLES, it is important to have a multi-disciplinary team working on the development. Our team was composed of healthcare professionals with much experience in stroke rehabilitation research including physical therapists, nurses as well as engineers and computer scientists who are experts in the field of technical innovation. Theoretical underpinnings through the use of the MRC framework and the human-centred design are highly important since both provide input and feedback from future users, such as stroke survivors, their caregivers and healthcare professionals, to the team. The research team used an evidence-based approach to developing ActivABLES, which provided knowledge about the potential for innovations to motivate and encourage stroke survivors to engage in home-based exercise and physical activity. Comprehensive and robust methods were used to conduct the study to gain a broad and extensive idea of the feasibility of ActivABLES among the participants and strong agreements were found between the findings based on the quantitative and qualitative data. Future studies investigating the effects of ActivABLES, should be conducted with larger samples and should investigate both short-term and long-term effects of ActivABLES on functional outcomes, as well as cost-effectiveness.

#### Clinical implications

Stroke survivors need to engage in exercise and physical activity to maintain and improve their function and independence in activities of daily living. Despite the importance of exercise and physical activity for stroke survivors, physical inactivity and sedentary behaviour is a major issue affecting community-dwelling stroke survivors. There is an urgent need to find ways to motivate stroke survivors to engage in exercise and physical activity on a daily basis with support from their caregivers and under the supervision of a physical therapist or nurses. Daily access to a physical therapist and other healthcare professionals is not possible and should not be necessary if the stroke survivors have other types of resources to promote own health in their homes. Use of ActivABLES in the home was found to be feasible by community-dwelling stroke survivors and their caregivers. In the future, ActivABLES may also be used in a broader context such as with stroke survivors residing in nursing homes, other patients and the elderly. We foresee ActivABLES as a low-cost technical solution which requires only a small space. The tools are not complicated to use and should not be expensive to produce.

Technical applications, like ActivABLES, have the potential to improve function in stroke survivors who reside in their homes since they encourage physical activity and self-initiated activities. Technical applications can offer games and feedback that motivate stroke survivors, helping them to engage in healthy behaviour. Stroke survivors can use technical applications for home-based exercise and physical activity, and they can be a resource to meet demand for follow-up service. Stroke survivors with slight to moderate impairments could possibly be self-reliant with technical applications that are simple and easy to use, provided that they are free of technical problems.

Technical solutions will be an increasing part of rehabilitation in the future but research has shown lack of confidence and competence of healthcare professionals in using those solutions [94]. Therefore, it is important to integrate use of technical resources into healthcare professionals’ education as well as the support given by healthcare organisations. Stroke survivors with slight or moderate handicap and their caregivers need appropriate resources to be more active in healthy behaviour in the community. In this way, stroke survivors can be empowered and take more initiative in their exercise and physical activities.

## Conclusion

There are many possibilities to encourage and help stroke survivors to be more physically active. ActivABLES is an intervention aiming to motivate and promote home-based exercise and physical activity of community-dwelling stroke survivors with support from their caregivers. The results from this feasibility study indicate that an interactive technical solution like ActivABLES is feasible to use and can be a good asset for stroke survivors with slight or moderate handicap to use in their homes. These results are encouraging for the researchers to further develop the prototypes of ActivABLES.

## Data Availability

Further information on the datasets is available from the corresponding author on reasonable request.

## Abbreviations

5xSST: Five Times Sit to Stand Test
ABC: Activities-Specific Balance Confidence Scale
BBS: Berg Balance Scale
BBT: Box and Block Test
BREQ-2: Behaviour Regulation Exercise Questionnaire 2
MRC: Medical Research Council
PT: Physical therapist
RN: Registered nurse
SDT: Self-determined theory
TUG: Timed-Up-and-Go
